# Cytokines as the good predictors of refractory *Mycoplasma pneumoniae* pneumonia in school-aged children

**DOI:** 10.1038/srep37037

**Published:** 2016-11-11

**Authors:** Yuanyuan Zhang, Shufen Mei, Yunlian Zhou, Meixia Huang, Guijuan Dong, Zhimin Chen

**Affiliations:** 1 Department of Pulmonology, Children’s Hospital, Zhejiang University School of Medicine, Hangzhou 310051, P.R. China; 2 Departement of Pediatrics, Red Cross Hospital of Hangzhou, 310003, China

## Abstract

Excessive immune response against pathogens may play an important role in refracory *Mycoplasma pneumoniae* pneumonia (RMPP). The aim of this study was to elucidate the associations between cytokines and the prediction of RMPP in school-aged patients. Retrospective analysis was performed on school-aged children with *Mycoplasma pneumoniae* pneumonia (MPP) hospitalized in our hospital between January 1, 2011 and December 31, 2015. The clinical charcteristics, including the cytokines in serum between the RMPP group and the general *Mycoplasma pneumoniae* pneumonia (GMPP) group were compared and the predictive values of RMPP were explored. Of total 180 patients, 115 patients were in the GMPP group, 65 were in the RMPP group. We found the levels of cytokines, including nterleukin (IL)-6, IL-10, interferon gamma (IFN-γ) in RMPP group were significantly higher than those in GMPP group (P < 0.01). In ROC curve analysis, IL-10 and IFN-γ were useful for differentiating patients with RMPP from those with GMPP. Logistic regression analysis showed that the IL-10 ≥ 3.65 pg/ml and IFN-γ ≥ 29.05 pg/ml were significant predictors regarding to RMPP. Additionally, a positive correlation between serum IL-10 and IFN-γ concentrations was observed. Conclusions: IL-10 and IFN-γ could be used as the good predictors of RMPP in school-aged children.

*Mycoplasma pneumoniae* (MP) is one of the major pathogens causing community-acquired pneumonia (CAP) in children^1^,^2^. MP was detected in 30% of pediatric CAP, and in over 50% among children aged 5 years or older^3^. Although *Mycoplasma pneumoniae* pneumonia (MPP) is usually considered as a self-limited disease, sometimes it may cause refractory *Mycoplasma pneumoniae* pneumonia (RMPP) showing clinical and radiological deterioration despite of macrolide antibiotic therapy for 7 days or longer^4^,^5^, and develop into a severe life-threatening pneumonia^6^,^7^.

Although the underlying mechanisms are still uncertain, the macrolide-resistant MP infection and excessive immunological inflammation are most commonly proposed^8^,^9^. In China, macrolide-resistant MP is very common and the prevalence ranges from 83% to 95%^10^,^11^. In our previous study, the prevalence of macrolide-resistance in MPP patients reached to 87.7%, and there were no significant difference of resistance rate of MP between the general MPP (GMPP) group and the RMPP group^10^. So excessive immune response against pathogens, such as vigorous expression of cytokines and highly activated cell-mediated immune response may play a more important role in RMPP than macrolide resistance in China. Recent clinical researches demonstrated that corticosteroids have been used with satisfactory therapeutic effect for children with RMPP^5^,^11^,^12^, which is suggestive of an overreaction of host immune systems. However, further studies are needed to elucidate the associations between excessive immune response, especially the cytokines and the progression of RMPP.

In the present study, the clinical characteristics of school-aged children diagnosed as RMPP were compared with school-aged children diagnosed as GMPP, including the cytokines in serum, and the predictive values of RMPP were explored.

## Results

### Clinical characteristics of RMPP and GMPP

A total of 180 children were recruited into this study, 115 patients were in the GMPP group, and the remaining 65 patients were in the RMPP group. These patients’ relevant demographic, clinical, laboratory and radiological data are presented in Table 1. In short, there were no significant differences between the two groups with respect to age, gender distribution, duration of symptom before admission and length before macrolide therapy. However, the median duration of fever and the median length of stay were significant longer in the RMPP group than those in the GMPP group (P < 0.01). And the incidence of extra-pulmonary complications was 27.7% in RMPP group and 8.7% in GMPP group, with a significant difference (P < 0.01).

Regarding the laboratory examinations, the median levels of CRP, LDH, and the median percentage of peripheral neutrophils in children with RMPP were significant higher than those in children with GMPP (P < 0.01), although no significant differences were observed in the median values of WBC, immunoglobulin and subtypes of T lymphocytes between the two groups. With regard to radiological findings, 83.1% of the patients in the RMPP group showed large lesions versus 53.9% in the GMPP group (P < 0.01). Furthermore, there was significant differences between the two groups in the incidence of pleural effusion (48.3% versus 11.0%, P < 0.01).

### Comparison of the serum cytokines between the RMPP group and the GMPP group

**Figure 1:**
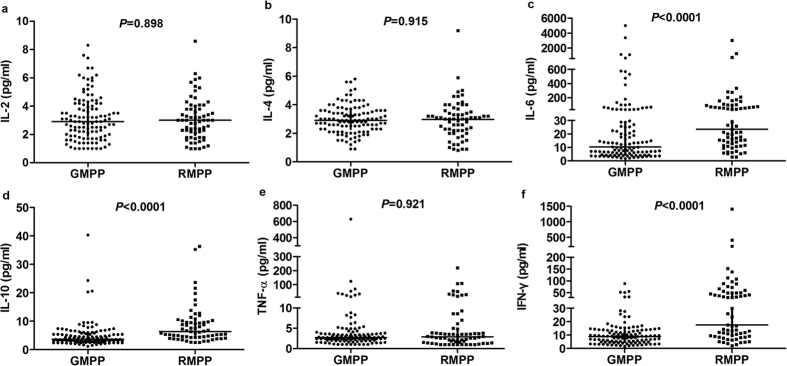
Comparison of serum cytokine concentrations between the GMPP group and the RMPP group. (**a**) IL-2; (**b**) IL-4; (**c**) IL-6; (**d**) IL-10; (**e**) TNF-a; (**f** ) IFN-γ.

A comparison of the serum cytokines between the RMPP group and the GMPP group is presented in Figure 1. In detail, the concentrations of serum IL-6 (median 23.40 pg/ml, 25^th^–75^th^ interquartile ranges 12.20~93.35 pg/ml), IL-10 (median 6.40 pg/ml, 25^th^–75^th^ interquartile ranges 4.35~10.00 pg/ml) and IFN-γ (median 17.50 pg/ml, 25^th^–75^th^ interquartile ranges 9.05~50.20 pg/ml) were significantly higher in patients with RMPP than in those with GMPP (Fig. 1c,d,f). However, serum IL-2, IL-4 and TNF-a concentrations did not differ significantly between the RMPP group and the GMPP group (P = 0.898, P = 0.915, P = 0.921, respectively) (Fig. 1a,b,e).

### Receiver operator characteristic (ROC) curves

**Figure 2:**
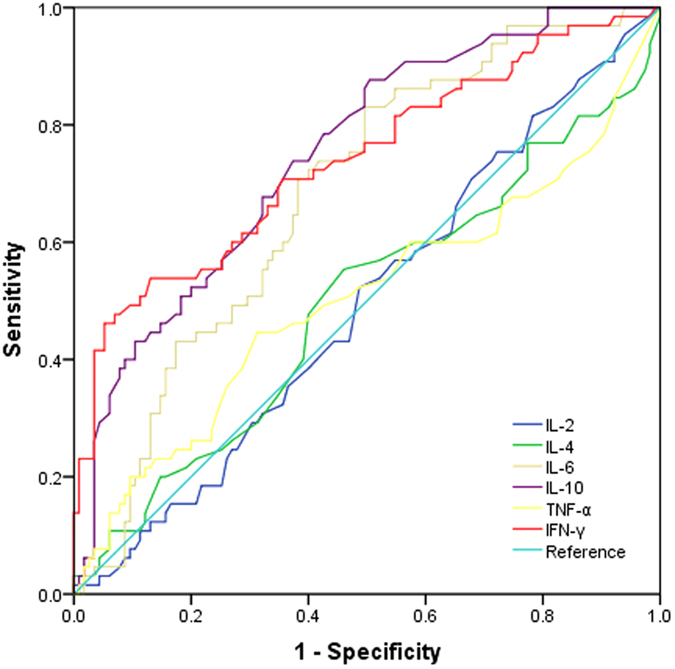
Receiver operator characteristic curves for differentiating RMPP from GMPP.

To explore the predictive values of cytokines, especially IL-6, IL-10 and IFN-γ, as biomarkers for RMPP, ROC curves were made and the cut-off values with maximum sensitivities and specificities were determined. Analysis of these ROC curves showed that the IL-10 and IFN-γ were useful for differentiating patients with RMPP from those with GMPP (Table 2 and Fig. 2). When the cut-off values for the IL-10 and IFN-γ were set at 3.65 pg/ml and 29.05 pg/ml, respectively, the sensitivity and specificity in differentiating RMPP from GMPP were 87.7% and 49.6%, 46.2% and 94.8%, respectively.

### Stepwise logistic regression

IL-10 and IFN-γ were analyzed in a stepwise logistic regression model. The results showed that serum IL-10 (odds ratio of 3.700, P = 0.003) and IFN-γ (odds ratio of 9.630, P = 0.000) were significant risk factors for RMPP (Table 3). Furthermore, serum IL-10 and IFN-γ concentrations showed a positive correlation (r = 0.578, P < 0.001).

## Discussion

MP is one of the major pathogens causing CAP in children, especially in school-aged children. MP infection was traditionally thought to be a self-limited process, but more and more RMPP, which display clinical and radiological progression after macrolide therapy for 7 days or longer, were reported in recent years^5^,^11^,^12^. The reasons why RMPP occurred remain unclear, but it is widely accepted that immunological response seemed to play an important role in the progress of RMPP^8^,^9^,^13^,^14^,^15^. In our other study, we found that the incidence of RMPP in school-aged children was more common than younger children (data not published). Some studies^8^,^16^ also found that children with RMPP were significant older (the mean age was 66.6~73.2 months) than children with GMPP. So age should be one of confounders of some clinical presentation and biological markers because children were in the process of growing. To our knowledge, until now this is the first study focused on the clinical characteristics of school-aged children diagnosed as RMPP and GMPP, and tried to elucidate the associations between excessive immune response, especially the cytokines and the progression of RMPP, which could avoid the statistic bias from age.

In the present, retrospective study, 180 patients with MPP were enrolled, 65 cases were diagnosed as RMPP, while 115 were GMPP. No distinctive differences of age, gender distribution, duration of symptom before admission and length before macrolide therapy were observed between the two groups, which meant the clinical course between the two groups were relatively consistent. However, longer duration of fever, longer length of stay, and higher incidence of extra-pulmonary complications were found in the RMPP group than those in the GMPP group, which indicated that to some extent children with RMPP had a more severe illness. With respect to radiological findings, pleural effusion and large lesions were more frequently found in the RMPP group than those in the GMPP group, which were similar to previous reports^8^,^16^.

Cell-mediated immunological response plays an important role in the progression of MPP. Several studies demonstrated that inflammatory cytokines were involved in the immunopathogenesis of MP infection^13^,^17^,^18^,^19^. We thought the cytokines might be better than LDH to reflect the immune response, though some research showed that the serum level of LDH could be a marker for RMPP^16^. In the present study, we focus on the serum cytokines, which were commonly detected in children with MPP in our hospital. Of interest, we found the concentrations of serum IL-6, IL-10, IFN-γ in the RMPP group were significant higher when compared with the GMPP group. These results indicated the RMPP might lead to release higher cytokines, such as IL-6, IL-10, IFN-γ, and then contribute to the excessive inflammation reaction.

To explore the predictive values of cytokines for RMPP, ROC curve analysis was made. In our study, we found that the area under the curve for IL-10 and IFN-γ were above 0.7 in ROC curve analysis, indicating fair discriminative power for predicting RMPP. The optimal cutoff value for IL-10 and IFN-γ was 3.65 pg/ml and 29.05 pg/ml, respectively. This indicates its clinical utility in identifying patients at high risk for RMPP. Our previously study^20^ showed that IL-6 ≥ 14.75 pg/ml was significant predictor regarding RMPP. However, in this study, in order to avoid the statistic bias from age we focused on the school-aged children whose immune system were relative mature, thus lead to the different values between this study with our previous study. Stepwise logistic regression analysis further confirmed that the IL-10 and IFN-γ were significant predictors for RMPP. Interactions among different cytokines are also an important aspect of the complex regulatory network of the immune response. Here, we observed a positive correlation between IL-10 and IFN-γ concentrations in the RMPP patients.

This study has several limitations. Firstly, it was a retrospective study, and therefore there may have been some selection bias. Secondly, the distribution of patients between the two groups is not matching, which might affect the statistic results. Thirdly, though we tried our best to exclude co-infection by performing examinations which could be carried out in our hospital, there may be some patients who had a combined MP and other pathogens infection which cannot be detected. Fourthly, we had not used any severity scoring system for children because of retrospective nature, which could not reflect the relationship between the increased level of cytokines and the underlying degree of sickness. However, despite these limitations, this present study is the first report focus on the school-aged children with MPP, and clearly indicated IL-10 and IFN-γ could be used as the good predictors of RMPP in school-aged children. Furthermore a prospective study enrolled with a large number of school-aged patients with RMPP is needed to be carried out to identify the potential utility of IL-10 and IFN-γ as the good predictors.

## Methods

### Case selection

This retrospective study was performed at Children’s hospital, Zhejiang University School of Medicine between January 1, 2011 and December 31, 2015. We enrolled school-aged patients if they had signs and symptoms indicative of pneumonia on admission, including fever, cough, abnormal lung auscultation and a new infiltrate on chest radiograph^12^, and were confirmed with MP infection. Patients who were co-infected with other pathogens were excluded in this study. Patients who received corticosteroids before admission or had underlying diseases such as asthma, recurrent respiratory tract infection, chronic cardiac and pulmonary disease, rheumatic diseases and immunodeficiency were also excluded. X-rays’ diagnosis were made based on the consultation from radiologists and respiratory physicians. The diagnosis of MP infection was based on the positive results for serologic test (MP IgM positive and antibody titer ≥1:160) while having the positive results for MP polymerase chain reaction (PCR) tests of nasopharyngeal aspirate/swab. Real-time PCR, using MP PCR kit (Daan Gene Ltd Co., Guangzhou, China), was performed on a 7500 Real-Time PCR System (Applied Biosystems) to detect MP DNA. Meanwhile, Antibodies to MP were measured using ELISA kit (Daan Gene Ltd Co., Guangzhou, China). The diagnosis of RMPP was based on the presence of persistent fever and clinical as well as radiological deterioration after azithromycin treatment for 7 days or longer^4^,^5^. To exclude other respiratory tract infections, all patients were performed protein purified derivative (PPD), microbiological testing of blood, pleural effusion and nasopharyngeal aspirate/swab cultures, nasopharyngeal aspirate/swab for virus antigens detection (respiratory syncytial viruses, influenza viruses, metapneumovirus, adenovirus, and parainfluenza virus), and serology for Chlamydia pneumoniae (CP), Chlamydia trachomatis (CT) and Legionella pneumophila (LG). None of the tests resulted in detection of any other pathogens.

### Study evaluation

During hospitalization demographic, clinical information, laboratory data and radiological findings from all children who were included in the study were retrospectively collected. Nasopharyngeal aspirate/swab specimens were routinely tested within 24 hours of admission for bacterial culture, virus using direct immunofluorescence assays and MP using PCR. Peripheral blood samples were obtained on admission for the determination of the culture, the white blood cell (WBC), C-reactive protein (CRP), lactate dehydrogenase (LDH), immunoglobulins, subpopulations of T lymphocytes, specific antibody to MP, CT, LG, and cytokines including interleukin (IL)-2, IL-4, IL-6, IL-10, tumor necrosis factor alpha (TNF-α) and interferon gamma (IFN-γ). Chest radiography was performed before admission or during hospitalization using standard equipment and radiographic techniques. Large lesion was defined when the extent of infiltration on chest radiography was more than 1/3 of the lung^21^. All patients were treated initially with macrolides (azithromycin or erythromycin) for 5–7 consecutive days or partly with beta-lactam antibiotics (ampicillin with sulbactam or ceftriaxone) simultaneously. All children enrolled were followed until discharging. Body temperature, extra-pulmonary complications^10^ and respiratory tract signs and symptoms of patients were examined at study entry and every 8 hour thereafter.

### Measurement of serum cytokines

The concentrations of IL-2, IL-4, IL-6, IL-10, TNF-α and IFN-γ in serum were determined using a CBA Human Th1/Th2 Cytokine Kit II (BD Biosciences, San Diego, CA, USA) according to the manufacturer’s specifications. Following the acquisition of sample data using a FACScalibur flow cytometer (BD Biosciences), results were generated using BD CBA Software (BD Biosciences, San Jose, CA, USA). Then we established the standard curve for each reagent. 1.0 pg/mL was the lowest detection limit for these six cytokines, while the highest was 5000 pg/mL. (The standard curve could be seen in the Supplementary Information).

### Approvals

All experiments were performed following the relevant guidelines and regulations of the Children’s Hospital, Zhejiang University School of Medicine. The methods were carried out in accordance with the approved guidelines. The study was approved by the ethics committee of the Children’s Hospital, Zhejiang University School of Medicine. During the follow-up period, written informed consent was obtained from at least one guardian of each patient who was included in the study.

### Statistical Analysis

Statistical analyses were performed using SPSS software (version 15.0). Normal distribution data were expressed as mean ±SD (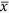 ± s). Independent-Samples T-test was used to compare these data. Skewed distribution data were expressed as median values (25^th^–75^th^ interquartile ranges). The comparisons were made by the Mann-Whitney U-test. And Chi-squared tests were used to compare categorical data. Receiver operating characteristic (ROC) curves were operated to evaluate candidate indicators with regards to the refractory assessment of patients with MMP. Logistic regression analysis was performed to select the variables associated with the RMPP. Statistical significance was defined as P < 0.05.

## Additional Information

**How to cite this article**: Zhang, Y. *et al*. Cytokines as the good predictors of refractory *Mycoplasma pneumoniae* pneumonia in school-aged children. *Sci. Rep*. **6**, 37037; doi: 10.1038/srep37037 (2016).

**Publisher’s note:** Springer Nature remains neutral with regard to jurisdictional claims in published maps and institutional affiliations.

## Supplementary Material

Supplementary Information

## Figures and Tables

**Table 1:** Comparison of clinical and laboratory variables between GMPP and RMPP.

Clinical and laboratory variables	GMPP (n = 115)	RMPP (n = 65)	*P*-value
Age, years	7.7 (6.6~9.7)	7.8 (6.9~9.9)	0.543
Sex (male/female)	62/53	26/39	0.088
Duration of symptom before admission, days	10 (8~12)	10 (8~12)	0.764
Length before macrolide therapy, days	5 (3~6)	4 (3~6)	0.124
Duration of fever, days	9 (6~11)	14 (12~17)	0.000
Length of stay, days	5 (3~7)	8 (7~10)	0.000
Extra-pulmonary complications	10 (8.7%)	18 (27.7%)	0.001
White blood cell (×10^9^/L)	6.92 (5.58~9.19)	7.60 (6.13~9.28)	0.287
Neutrophil, %	65.2 (56.2~70.8)	75.9 (69.7~80.0)	0.000
C-reactive protein, mg/L)	11 (4~28)	52 (19~90)	0.000
Lactatedehydrogenase (LDH), IU/L	337 (265~440)	520 (401~661)	0.000
Total Immunoglobulin (Ig), g/L			
IgG	11.20 (9.60~13.76)	10.40 (8.68~12.64)	0.115
IgA	1.50 (1.11~2.07)	1.49 (1.07~1.92)	0.932
IgM	1.55 (1.19~2.11)	1.85 (1.21~2.32)	0.226
Subtypes of T lymphocytes, %			
CD3^+^	63.27 ± 11.40	62.40 ± 12.35	0.646
CD4^+^	32.98 ± 9.83	33.64 ± 7.62	0.656
CD8^+^	24.20 ± 7.83	24.05 ± 7.28	0.910
Radiological features			
% Patients with large lesions	62 (53.9%)	54 (83.1%)	0.000
% Patients with Pleural effusion	26 (22.6%)	35 (53.8%)	0.000

Data are presented as the mean ± standard deviation, number (percentage), median (25^th^–75^th^ percentile). The laboratory data in table 1 were achieved from patients on the day of admission.

**Table 2:** ROC curve analysis for predicting RMPP in school-aged patients.

parameter	AUC	Cutoff value	Sensitivity	Specificity	P-value	95% confidence interval
IL-2, pg/ml	0.494	2.95	0.523	0.513	0.897	0.407~0.581
IL-4, pg/ml	0.495	2.95	0.554	0.539	0.913	0.404~0.586
IL-6, pg/ml	0.682	10.50	0.831	0.504	0.000	0.603~0.760
IL-10, pg/ml	0.751	3.65	0.877	0.496	0.000	0.679~0.823
TNF-α, pg/ml	0.504	3.45	0.446	0.687	0.921	0.410~0.599
IFN-γ, pg/ml	0.739	29.05	0.462	0.948	0.000	0.660~0.818

AUC: area under the ROC curve; Cut-off value: the value on the ROC curve is closest to the upper right to take maximum sensitivity and specificity; P-value: the AUC value of the independent factors compared to ROC curve reference value 0.5. The laboratory data in table 2 were achieved from patients on the day of admission.

**Table 3:** Stepwise logistic regression analysis for the related factors predicting the RMPP.

Variable	B	S.E.	Wald	P-value	OR	95% CI	
						Lower	Upper
IL-10 ≥3.65 pg/ml	1.308	0.446	8.608	0.003	3.700	1.544	8.865
IFN-γ ≥29.05 pg/ml	2.265	0.506	20.016	0.000	9.630	3.570	25.972
