# Supplementary material for: Cytokines as the good predictors of refractory Mycoplasma pneumoniae pneumonia in school-aged children
Source: Sci Rep. 2016 Nov 11;6:37037. doi: 10.1038/srep37037 (PMC5105141; doi:10.1038/srep37037)

Cytokines as the good predictors of refractory *Mycoplasma pneumoniae* pneumonia in school-aged children

Yuanyuan Zhang[1&], Shufen Mei[2&], Yunlian Zhou[1&], Meixia Huang[1], Guijuan Dong[1], Zhimin Chen[1*]

[1]Department of Pulmonology, Children's Hospital, Zhejiang University School of Medicine, Hangzhou 310051;

[2]Departement of Pediatrics, Red Cross Hospital of Hangzhou, 310003;

[&]contribute equally

*Correspondence to: Dr Zhimin Chen, Department of Pulmonology, Children's Hospital, Zhejiang University School of Medicine, No. 3333 Binsheng Road, Hangzhou 310051, P.R. China.

Tel: (+86-571)- 86670024, Fax: (+86-571)-87033296. E-mail: drchenzm@163.com.

	Calibration Files	IFN-γ						TNF-α					
		Concentration (pg/ml)	FL2 MFI	Logistic	R Numerator	(y-y_avg)^2	Optimize	Concentration (pg/ml)	FL2 MFI	Logistic	R Numerator	(y-y_avg)^2	Optimize
		x	y	y'				x	y	y'			
**1**	cba-0000-20.007	0.00	0.40	0.40	0.16	0.16	0.00	0.00	0.39	0.39	0.15	0.15	0.00
**2**	cba-0020a.003 copy	1.30	0.83	0.81	0.68	0.69	0.00	1.30	1.14	1.09	1.24	1.30	0.00
**3**	cba-0040d2.009 copy	1.60	1.01	1.04	1.06	1.03	0.00	1.60	1.32	1.34	1.77	1.73	0.00
**4**	cba-0080d1.010 copy	1.90	1.31	1.32	1.73	1.73	0.00	1.90	1.59	1.61	2.56	2.54	0.00
**5**	cba-0156d2.013 copy	2.19	1.64	1.61	2.64	2.68	0.00	2.19	1.86	1.87	3.48	3.45	0.00
**6**	cba-0312d1.014 copy	2.49	1.93	1.94	3.73	3.71	0.00	2.49	2.16	2.16	4.67	4.66	0.00
**7**	cba-0625d1.017 copy	2.80	2.27	2.27	5.15	5.15	0.00	2.80	2.45	2.46	6.02	5.99	0.00
**8**	cba-1250d2.019 copy	3.10	2.60	2.61	6.77	6.76	0.00	3.10	2.80	2.76	7.72	7.81	0.00
**9**	cba-2500d1.020 copy	3.40	2.94	2.93	8.62	8.63	0.00	3.40	3.09	3.07	9.47	9.53	0.00
**10**	cba-5000d1.022 copy	3.70	3.25	3.25	10.54	10.53	0.00	3.70	3.35	3.37	11.30	11.22	0.00

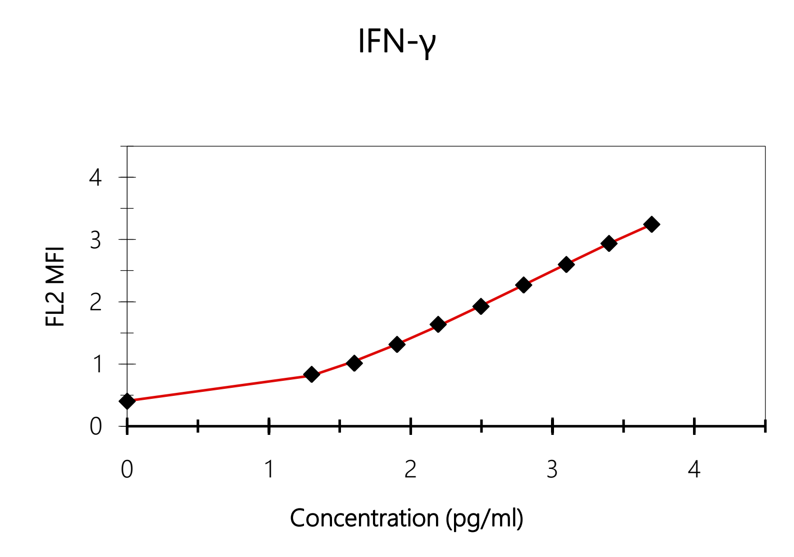

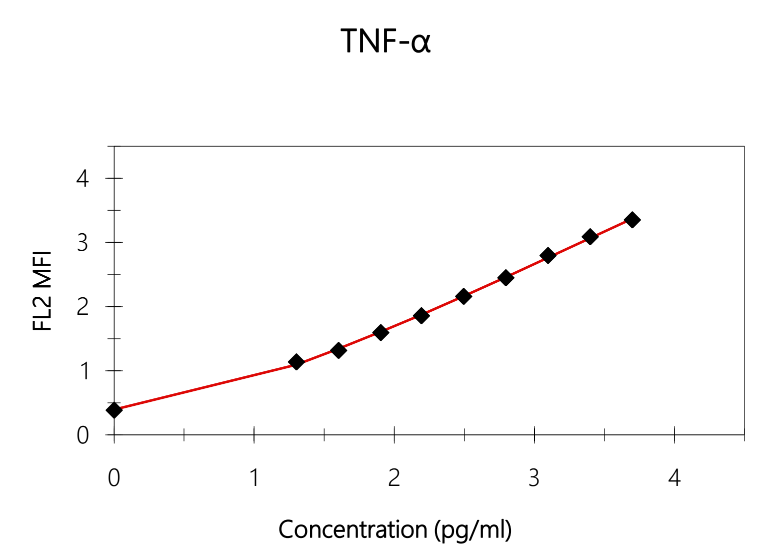

	Calibration Files	IL-10						IL-6					
		Concentration (pg/ml)	FL2 MFI	Logistic	R Numerator	(y-y_avg)^2	Optimize	Concentration (pg/ml)	FL2 MFI	Logistic	R Numerator	(y-y_avg)^2	Optimize
		x	y	y'				x	y	y'			
**1**	cba-0000-20.007	0.00	0.38	0.39	0.15	0.15	0.00	0.00	0.46	0.46	0.21	0.21	0.00
**2**	cba-0020a.003 copy	1.30	1.26	1.24	1.57	1.60	0.00	1.30	1.32	1.28	1.69	1.73	0.00
**3**	cba-0040d2.009 copy	1.60	1.51	1.51	2.28	2.27	0.00	1.60	1.53	1.54	2.36	2.33	0.00
**4**	cba-0080d1.010 copy	1.90	1.76	1.79	3.15	3.10	0.00	1.90	1.79	1.82	3.26	3.21	0.00
**5**	cba-0156d2.013 copy	2.19	2.08	2.06	4.30	4.34	0.00	2.19	2.09	2.09	4.37	4.35	0.00
**6**	cba-0312d1.014 copy	2.49	2.34	2.36	5.50	5.46	0.00	2.49	2.38	2.38	5.66	5.65	0.00
**7**	cba-0625d1.017 copy	2.80	2.64	2.65	6.99	6.95	0.00	2.80	2.69	2.67	7.20	7.25	0.00
**8**	cba-1250d2.019 copy	3.10	2.98	2.95	8.80	8.91	0.00	3.10	2.99	2.97	8.86	8.92	0.00
**9**	cba-2500d1.020 copy	3.40	3.25	3.25	10.56	10.56	0.00	3.40	3.26	3.26	10.62	10.61	0.00
**10**	cba-5000d1.022 copy	3.70	3.54	3.55	12.54	12.50	0.00	3.70	3.54	3.55	12.57	12.53	0.00

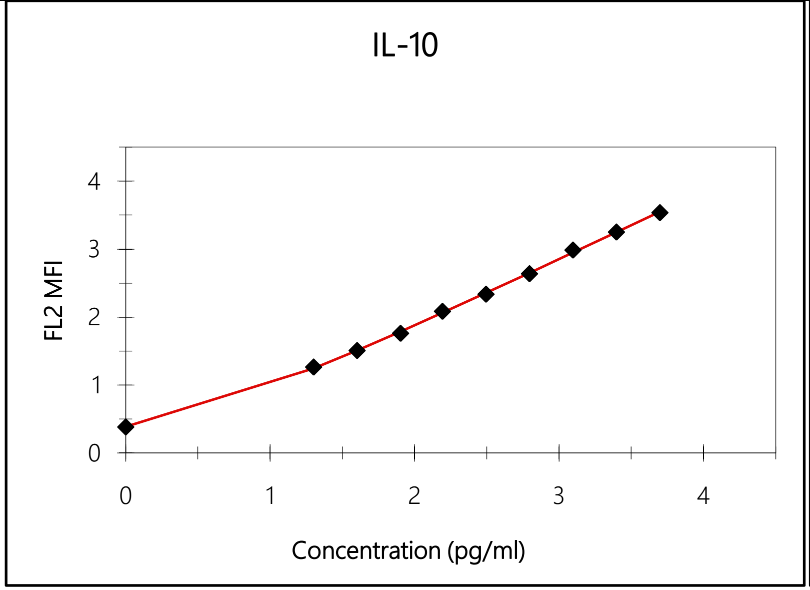

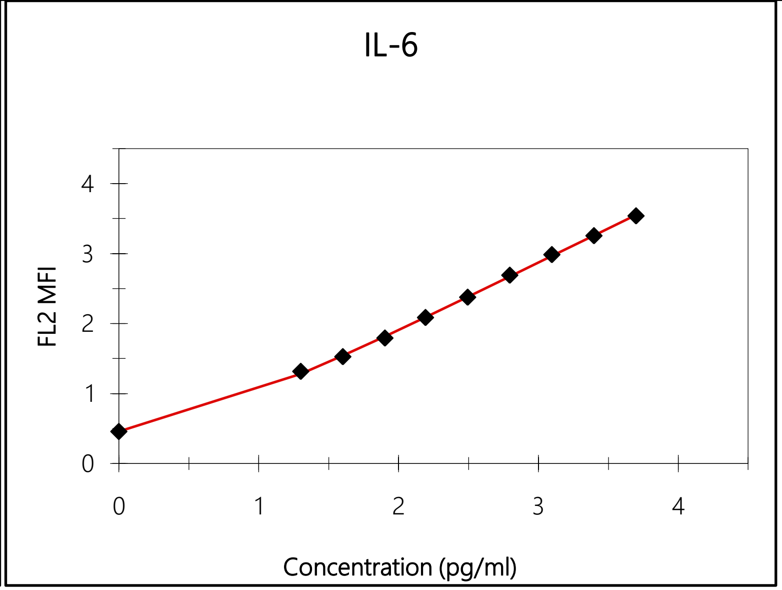

	Calibration Files	IL-4 Concentration (pg/ml) x	FL2 MFI y	Logistic y'	R Numerator	(y-y_avg)^2	Optimize
1	cba-0000-20.007	0.00	0.42	0.42	0.17	0.17	0.00
2	cba-0020a.003 copy	1.30	1.17	1.16	1.35	1.36	0.00
3	cba-0040d2.009 copy	1.60	1.43	1.41	2.02	2.05	0.00
4	cba-0080d1.010 copy	1.90	1.67	1.69	2.81	2.77	0.00
5	cba-0156d2.013 copy	2.19	1.95	1.96	3.84	3.82	0.00
6	cba-0312d1.014 copy	2.49	2.25	2.26	5.08	5.04	0.00
7	cba-0625d1.017 copy	2.80	2.56	2.56	6.55	6.54	0.00
8	cba-1250d2.019 copy	3.10	2.91	2.87	8.37	8.49	0.00
9	cba-2500d1.020 copy	3.40	3.17	3.18	10.07	10.04	0.00
10	cba-5000d1.022 copy	3.70	3.49	3.49	12.16	12.15	0.00

	Calibration Files	IL-2 Concentration (pg/ml) x	FL2 MFI y	Logistic y'	R Numerator	(y-y_avg)^2	Optimize
1	cba-0000-20.007	0.00	0.39	0.40	0.16	0.16	0.00
2	cba-0020a.003 copy	1.30	1.22	1.14	1.40	1.49	0.01
3	cba-0040d2.009 copy	1.60	1.38	1.42	1.97	1.92	0.00
4	cba-0080d1.010 copy	1.90	1.67	1.71	2.86	2.79	0.00
5	cba-0156d2.013 copy	2.19	1.97	2.01	3.97	3.90	0.00
6	cba-0312d1.014 copy	2.49	2.34	2.33	5.44	5.46	0.00
7	cba-0625d1.017 copy	2.80	2.67	2.65	7.08	7.14	0.00
8	cba-1250d2.019 copy	3.10	3.00	2.97	8.92	9.02	0.00
9	cba-2500d1.020 copy	3.40	3.30	3.29	10.86	10.91	0.00
10	cba-5000d1.022 copy	3.70	3.57	3.60	12.84	12.72	0.00

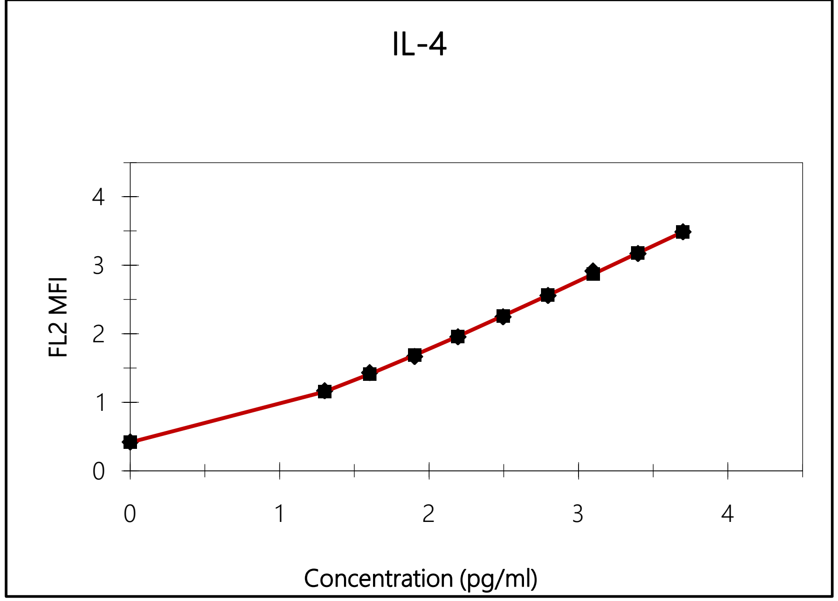

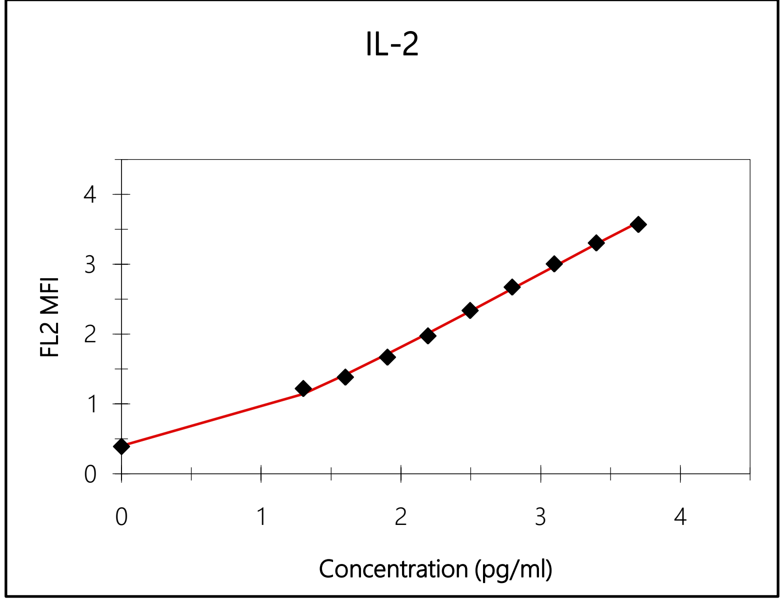

Supplement: Supplementary Information [file srep37037-s1.pdf]
